# Inflammation-Linked Muscle Atrophy in Limb Girdle Muscular Dystrophy R1 (LGMDR1): Insights into Disease Mechanisms

**DOI:** 10.3390/cimb48040361

**Published:** 2026-03-30

**Authors:** Sukanya Banerjee, Bishan Dass Radotra, Manni Luthra-Guptasarma, Manoj K. Goyal

**Affiliations:** 1 Department of Histopathology, Post Graduate Institute of Medical Education and Research, Chandigarh 160012, India; sbanerjee2808@yahoo.com; 2 Department of Immunopathology, Post Graduate Institute of Medical Education and Research, Chandigarh 160012, India; mguptasarma@yahoo.com; 3 Department of Neurology, Post Graduate Institute of Medical Education and Research, Chandigarh 160012, India; goyal_mk@yahoo.com

**Keywords:** LGMDR1, inflammation, TNF-α signaling, muscle atrophy, ubiquitin-proteasome system

## Abstract

Background: Muscle atrophy is a major feature of Limb Girdle Muscular Dystrophy R1 (LGMDR1) patients, but its underlying molecular mechanisms have not been fully explored. While the ubiquitin–proteasome system (UPS) is known to be involved in muscle protein degradation, inflammation commonly observed in LGMDR1 patients may further activate the UPS. This study aimed to explore the role of inflammation in the muscle atrophy of LGMDR1 patients. Methods: Muscle biopsies from six confirmed LGMDR1 patients (with *CAPN3* variants and reduced calpain-3 protein expression) were analyzed for atrophy-related markers, MuRF1 and Atrogin-1, using qRT-PCR and Western blotting. The expression of cytokines, TNF-α, IL-1β, and IL-6 was analyzed by qRT-PCR from muscle biopsies and by ELISA from serum samples. The *NFκB*, *FOXO1*, and *FOXO3* gene expression was analyzed using qRT-PCR and Western blotting from muscle biopsies. Results: Elevated TNF-α levels were associated with increased UPS activity, reflected by upregulated NFκB, FOXO1, MuRF1, and Atrogin-1 expression in LGMDR1. Conclusion: Our findings indicate that increased TNF-α expression is associated with muscle wasting in LGMDR1 patients by targeting UPS pathway mediators that activate ubiquitin ligases—MuRF1 and Atrogin-1. These findings suggest that targeting TNF-α signaling and its downstream factors may help develop therapeutic interventions to prevent muscle atrophy in LGMDR1 patients.

## 1. Introduction

Limb Girdle Muscular Dystrophy R1 (LGMDR1) is a progressive neuromuscular disorder resulting from variants in the *CAPN3* gene, which leads to calpain-3 protein deficiency [1,2]. A recent molecular genetics study has highlighted the genetic diversity of limb-girdle muscular dystrophies and their clinical overlap with other muscle disorders [3]. Recent large-scale datasets have confirmed *CAPN3* mutation hotspots and provided insights into the broader genetic landscape of LGMDR1 [4]. Another recent study has identified novel splicing and insertion variants in *CAPN3* using targeted sequencing, further expanding the mutation spectrum of LGMDR1 [5]. Dysfunction of calpain-3 impairs muscle fiber cytoskeletal remodeling and disrupts protein signaling regulation [6]. In addition, calpain-3 is involved in sarcomere organization, calcium regulation, and muscle repair [7]. It is the most common subtype of LGMD, accounting for 26.5–30% of all LGMDs [8]. A population-based study in a Czech cohort reported that LGMDR1 accounted for over 50% of genetically confirmed LGMD cases [9]. The clinical features of LGMDR1 include progressive proximal muscle degeneration of the pelvic and shoulder girdle [10], resulting in muscle weakness and atrophy, often accompanied by bilateral scapular winging and Achilles tendon contracture [11]. Experimental *CAPN3*-deficient mouse models have shown that loss of *CAPN3* function leads to metabolic imbalance and systemic energy dysregulation, along with progressive muscle pathology [12]. Histopathological analyses suggest that calpainopathy shows a characteristic inflammatory profile that differs from classical inflammatory myopathies [13]. Moreover, calpainopathy patients may be histopathologically misdiagnosed as inflammatory myopathy, particularly with elevated creatine kinase levels and proximal muscle weakness [14]. Recent research has suggested that *CAPN3*-related muscular dystrophy patients with clinical heterogeneity may show asymptomatic hyperCKemia, exertional myalgia, or mild proximal weakness [15]. Additionally, a recent study on different myopathies has shown that muscle degeneration may be due to chronic inflammation, mitochondrial dysfunction, and impaired calcium signaling [16].

Muscle atrophy is driven by an imbalance between the dynamic anabolic and catabolic stimuli, where muscle protein breakdown far exceeds protein synthesis [17]. Skeletal muscle atrophy is also due to impaired muscle regeneration, dysregulation of mitochondrial function, the ubiquitin–proteasome system (UPS), and the autophagy–lysosome pathway [18]. The ubiquitin–proteasome system and autophagy–lysosome pathway are the two main intracellular pathways associated with protein degradation in eukaryotic cells [19]. The recent literature has highlighted that muscle proteostasis is maintained through the coordinated interaction of these two pathways during muscle loss [20,21]. Impairment of either of the two pathways leads to the accumulation of damaged protein and progressive loss of muscle function [22]. The UPS pathway is the primary signaling pathway of muscle atrophy, in which proteins are degraded through the proteasome complex. Ubiquitin ligases cause protein degradation by activating the 26S proteasome complex, which recognizes the ubiquitin chains attached to the protein as signals for degradation [23]. The lysine-48 (K48) linked polyubiquitin chain significantly targets the protein for degradation through the proteasomal complex [24]. MuRF1 (*TRIM63*) and Atrogin-1 (*FBXO32*), muscle-specific ubiquitin ligases, are consistently reported as critical markers across diverse muscle atrophic conditions [17]. Further, a recent meta-analysis has correlated Atrogin-1 and MuRF1 expression with decreased muscle fiber cross-sectional area [25]. The previous literature using a hindlimb unloading rat model has proven that skeletal muscle atrophy occurs due to inflammation [26,27]. An increased level of pro-inflammatory cytokines such as TNF-α, IL-1, and IL-6 activates NFκB signaling by degradation of IκBα through proteasome machinery [28,29]. Increased expression of NFκB sequentially activates MuRF1 [30,31] and Atrogin-1 [32], further stimulating muscle atrophy in mice. The molecular mechanism underlying muscle fiber atrophy in LGMDR1 is unclear. Calpain-3 deficiency in skeletal muscle is associated with mitochondrial abnormalities, energy deficit, and increased oxidative stress [33]. Further, oxidative stress and increased NFκB p65 expression have been implicated in the loss of muscle fibers in LGMDR1 patients, increasing protein ubiquitinylation. Thus, increased protein ubiquitinylation causes loss of skeletal muscle fibers in LGMDR1 or calpainopathy patients [34]. However, molecular mechanisms linking inflammation to muscle atrophy are not entirely understood. Interestingly, a study of cytokine (IL-1β, TNF-α, and IL-10) levels in LGMDR1 patients showed only a significant decrease in IL-10 levels in these patients [35]. Eosinophilic myositis is an additional feature observed in the histopathology of LGMDR1 patients [36], suggesting pro-inflammatory cytokines cause activation of the UPS pathway, leading to muscle atrophy in LGMDR1 patients.

Another leading regulator of muscle proteostasis is the transcription factor FOXO, which belongs to the superfamily of FOX regulatory proteins and can also play a role in the degradation of muscle proteins [37]. Phosphorylation of FOXO protein by AKT (Protein Kinase B) has been shown to inhibit the expression of FOXO, thereby allowing FOXO to remain in an inactive state in the cytoplasm [38,39,40]. Previous studies suggest that AKT suppresses the expression of Atrogin-1 and MuRF1 by inhibiting FOXO transcription factors [41,42]. The implication of the AKT-FOXO axis in muscle atrophy of LGMDR1 patients has not been previously explored.

In this study, we addressed the role of inflammation in regulating UPS in the context of LGMDR1. The ubiquitin–proteasome system is a key mediator of muscle protein breakdown. However, the link between UPS and inflammatory signaling in LGMDR1 remains unexplored. Our study highlighted the relationship between inflammatory cytokines and UPS activation, leading to muscle atrophy in LGMDR1 patients. This contributes to understanding disease pathogenesis and the development of new therapeutic strategies to attenuate it by modulating inflammation.

## 2. Materials and Methods

Patients with the clinical feature of muscular dystrophy with increased creatine kinase (CK) level and myopathic pattern on EMG were included in this study. The demographic and clinical records of LGMD patients were noted. Finally, we confirmed six LGMDR1 patients (out of 34 LGMD cases) based on *CAPN3* variants and reduced calpain-3 protein expression in Western blot [43], and included these 6 patients in this study. The clinical characteristics and *CAPN3* variants of these six patients are provided in Supplementary Table S1 [44]. Muscle biopsy samples were collected from the Department of Neurology. Muscle biopsies were primarily obtained from the biceps or deltoid muscles; however, in some cases, samples were also collected from the hamstring and vastus lateralis muscles. A vastus lateralis muscle sample free of any muscle disease was taken as a control (*n* = 5), obtained from the thigh flaps used during craniectomy procedures. Muscle biopsies were snap-frozen after collection and stored at −80 °C for further experiments. Serum was isolated from blood samples of LGMDR1 patients and stored at −80 °C. Informed consent was obtained from all the patients. The ethical clearance was obtained from the Institutional Ethics Committee (IEC) of the Post Graduate Institute of Medical Education and Research, Chandigarh (NK/4563/PhD).

### 2.1. RNA Isolation, cDNA Synthesis, and Quantitative Real-Time PCR from Muscle Biopsy of LGMDR1 Patients

Total RNA was extracted from snap-frozen muscle biopsies of LGMDR1 patients using miRvana Paris Kit (Invitrogen, Thermo Fisher Scientific, Waltham, MA, USA) (Invitrogen) according to the manufacturer’s instructions and quantified spectrophotometrically. The RNA was reverse-transcribed using 1 μg of total RNA in a total volume of 20 μL using the iScript cDNA synthesis kit (Bio-Rad Laboratories, Hercules, CA, USA). The cDNA prepared was stored at −20 °C for quantitative real-time PCR (qRT-PCR). Expression of *TRIM63*, *FBXO32*, *TNF*, *IL1B*, *IL6*, *NFkB (RELA)*, *FOXO1*, and *FOXO3* genes was determined by qRT-PCR using the ABI StepOne Plus Real-Time PCR (Applied Biosystems, Thermo Fisher Scientific, Waltham, MA, USA) and Takara TB Green Premix Ex Taq II (Takara Bio Inc., Shiga, Japan). The results of the qRT-PCR were normalized to the *GAPDH* gene (internal control) by the ΔΔ Ct method.

### 2.2. Western Blotting from Muscle Biopsy of LGMDR1 Patients

Total protein was extracted from snap-frozen muscle biopsy tissues with lysis buffer (50 mM Tris pH 7.5, 150 mM NaCl, 10 mM MgCl_2_, 1 mM EDTA, 10% glycerol, 2% SDS, 1% Triton X-100) [17] for MuRF1, Atrogin-1, FOXO1 protein, and with RIPA buffer for NFκB, IκBα, and AKT protein. Tissue was lysed in the presence of protease and phosphatase inhibitor, and the tissue lysate was centrifuged at 13,400 rpm for 15 min at 4 °C. The protein in the supernatant was quantified by the BCA assay (Thermo Fisher Scientific, Waltham, MA, USA). A total of 40 µg of protein was subjected to SDS-PAGE gel followed by semi-dry transfer. After this, membrane was blocked using 5% skimmed milk in 1× TBST for 1 h (For phospho antibody, membrane was blocked using 5% BSA in 1× TBST for 1 h) and then incubated with primary antibodies: Anti-MuRF1 antibody (Santa Cruz Biotechnology, Dallas, TX, USA; #398608); Anti-Fbx32 antibody (Abcam, Cambridge, UK; #168372); anti-NFκB antibody (Cell Signaling Technology, Danvers, MA, USA; #4764); anti-phospho-NFκB (Ser 536) antibody (Cell Signaling Technology, Danvers, MA, USA; #3033); anti-FOXO1 antibody (Cell Signaling Technology, Danvers, MA, USA; #2880); anti-IκBα antibody (Cell Signaling Technology, Danvers, MA, USA; #4814), anti-phospho- IκBα (Ser 32/36) antibody (Cell Signaling Technology, Danvers, MA, USA; #9246); anti-phospho-FOXO1 (Ser 256) antibody (Cell Signaling Technology, Danvers, MA, USA; #9461); anti-AKT antibody (Cell Signaling Technology, Danvers, MA, USA; #9272); anti-phospho-AKT (Ser 473) antibody (Cell Signaling Technology, Danvers, MA, USA; #9271) at 4 °C, overnight. The next day, the membrane was incubated with an appropriate secondary antibody for 1 h at room temperature and developed with chemiluminescence substrate (Biorad Clarity Western ECL substrate kit; Bio-Rad Laboratories, Hercules, CA, USA). GAPDH was used as a loading control. Analysis of Western blotting was done by densitometry using ImageJ software (version 1.54r, National Institutes of Health, Bethesda, MD, USA) (https://imagej.en.softonic.com/download; accessed on 27 January 2026).

### 2.3. Enzyme-Linked Immunosorbent Assay (ELISA) from Serum Samples of LGMDR1 Patients

Venous blood was collected from calpainopathy patients in serum separator tubes and allowed to clot at room temperature for 1 h. For isolation of serum, blood samples were centrifuged at around 1000× *g* for 5 min. The serum was collected in sterile microcentrifuge tubes and stored at −80 °C. These serum samples from calpainopathy patients were assayed for TNF-α, IL-1β, and IL-6 using Enzyme-Linked Immunosorbent Assay (ELISA) (Diaclone human ELISA kit (Diaclone SAS, Besançon, France)) according to the manufacturer’s instructions. For ELISA, standard vials were reconstituted with the standard diluent volume, giving a stock solution of 800 pg/mL of TNF-α, 500 pg/mL of IL-1β, and 200 pg/mL of IL-6. Serial dilutions of the standard were made directly in the assay plate to provide the concentration range. Absorbance was recorded at 450 nm, and cytokine levels were measured using corresponding cytokine standards.

### 2.4. Statistical Analysis

Data analysis was performed using GraphPad Prism statistical software (version 10). Normal distribution of data was analyzed by the Shapiro–Wilk test. Non-parametric data were analyzed by the Mann–Whitney test between two groups, and parametric data were analyzed by the unpaired *t*-test with Welch’s correction. The difference between the two groups was considered significant, where the *p*-value was <0.05. The number of samples used for each experiment is summarized in Supplementary Table S2.

## 3. Results

### 3.1. Expression of MuRF1 and Atrogin-1 by qRT-PCR and Western Blotting from Muscle Biopsy of Calpainopathy Patients

**Figure 1:**
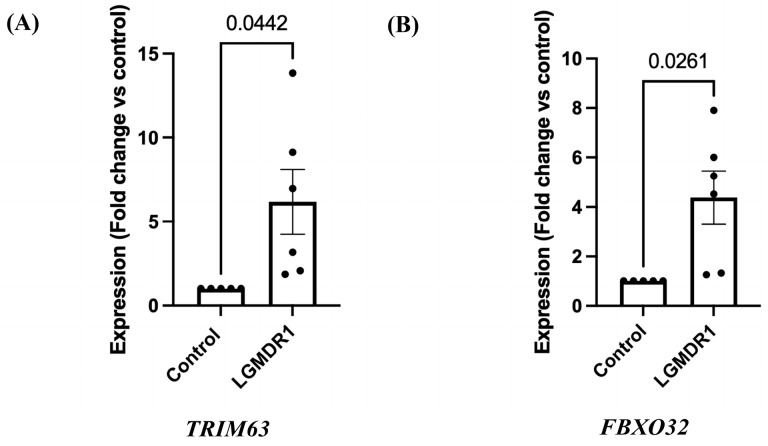
Expression of *TRIM63* and *FBXO32* genes in LGMDR1 patients by qRT-PCR. (**A**) Column bar graph showing significant upregulation of *TRIM63* gene expression in LGMDR1 patients (*p* = 0.0442, unpaired *t*-test with Welch’s correction). Data are mean ± SEM and shown as fold change relative to control (LGMDR1, *n* = 6; controls, *n* = 5). (**B**) Column bar graph showing significant upregulation of *FBXO32* gene expression in LGMDR1 patients (*p* = 0.0261, unpaired *t*-test with Welch’s correction). Data are mean ± SEM and shown as fold change relative to control (LGMDR1, *n* = 6; controls, *n* = 5).

**Figure 2:**
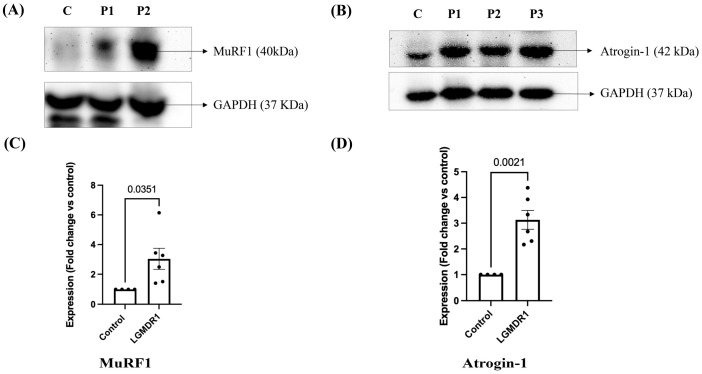
Expression of MuRF1 and Atrogin-1 proteins in LGMDR1 patients by Western blotting. (**A**) MuRF1 protein expression by Western blot, (**B**) Atrogin-1 protein expression by Western blot, C, control; P1-P3, individual LGMDR1 patient samples. (**C**) Column bar graph showing significant upregulation of MuRF1 protein expression in LGMDR1 patients (*p* = 0.0351, unpaired *t*-test with Welch’s correction). Data are mean ± SEM and shown as fold change relative to control (LGMDR1, *n* = 6; controls, *n* = 4). (**D**) Column bar graph showing significant upregulation of Atrogin-1 protein expression in LGMDR1 patients (*p* = 0.0021, unpaired *t*-test with Welch’s correction). Data are mean ± SEM and shown as fold change relative to control (LGMDR1, *n* = 6; controls, *n* = 4).

qRT-PCR (Figure 1) as well as protein expression (Figure 2) for muscle atrophy markers, *MuRF1* and *Atrogin-1,* were analyzed in six LGMDR1 patients. A statistically significant upregulation of *MuRF1* (*TRIM63*) gene (Figure 1A, *p* = 0.0442, unpaired *t*-test with Welch’s correction; 95% CI: 0.1965–10.16) was observed in patients compared to controls (*n* = 5). Similar observation was found for *Atrogin-1 (FBXO32)* gene (Figure 1B, *p* = 0.0261, unpaired *t*-test with Welch’s correction; 95% CI: 0.6002–6.152) compared to controls (*n* = 5).

Western blots showed intense bands of MuRF1 and Atrogin-1 at 40 and 42 kDa position respectively in LGMDR1 patients compared to controls (Figure 2A,B), with significantly increased expression of MuRF1 (*p* = 0.0351, unpaired *t*-test with Welch’s correction; 95% CI: 0.2114–3.868) (Figure 2C) and Atrogin-1 (*p* = 0.0021, unpaired *t*-test with Welch’s correction; 95% CI: 1.189–3.075) (Figure 2D) respectively in patients, as compared to controls (*n* = 4). Full-length uncropped Western blot images are provided in Supplementary Figure S1.

### 3.2. Expression of Pro-Inflammatory Cytokines in Calpainopathy Patients

**Figure 3:**
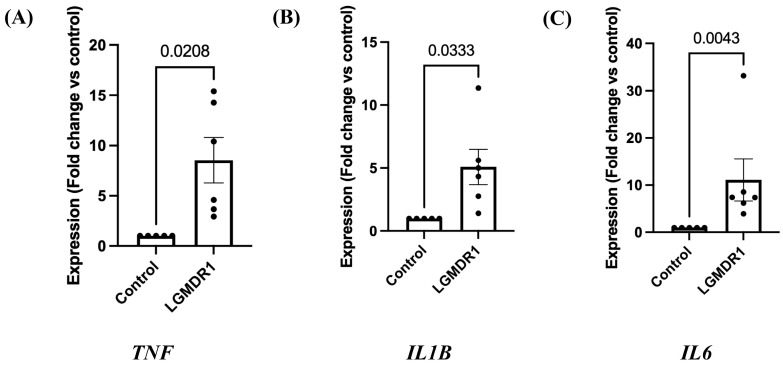
Expression of *TNF*, *IL1B*, and *IL6* genes in LGMDR1 patients by qRT-PCR. (**A**) Column bar graph showing significant upregulation of *TNF* gene expression in LGMDR1 patients (*p* = 0.0208, unpaired *t*-test with Welch’s correction). Data are mean ± SEM and shown as fold change relative to control (LGMDR1, *n* = 6; controls, *n* = 5). (**B**) Column bar graph showing significant upregulation of *IL1B* gene expression in LGMDR1 patients (*p* = 0.0333, unpaired *t*-test with Welch’s correction). Data are mean ± SEM and shown as fold change relative to control (LGMDR1, *n* = 6; controls, *n* = 5). (**C**) Column bar graph showing significant upregulation of *IL6* gene expression in LGMDR1 patients (*p* = 0.0043, Mann–Whitney test). Data are mean ± SEM and shown as fold change relative to control (LGMDR1, *n* = 6; controls, *n* = 5).

qRT-PCR of *TNF*, *IL1B*, and *IL6* genes was done in muscle biopsy of LGMDR1 patients. Significant upregulation of *TNF* (*n* = 6) (Figure 3A, *p* = 0.0208, unpaired *t*-test with Welch’s correction; 95% CI: 1.718–13.37), *IL1B* (*n* = 6) (Figure 3B, *p* = 0.0333, unpaired *t*-test with Welch’s correction; 95% CI: 0.4798–7.698) and *IL6* (*n* = 6) (Figure 3C, *p* = 0.0043, Mann–Whitney test) genes was found in calpainopathy patients compared to control subjects (*n* = 5).

**Figure 4:**
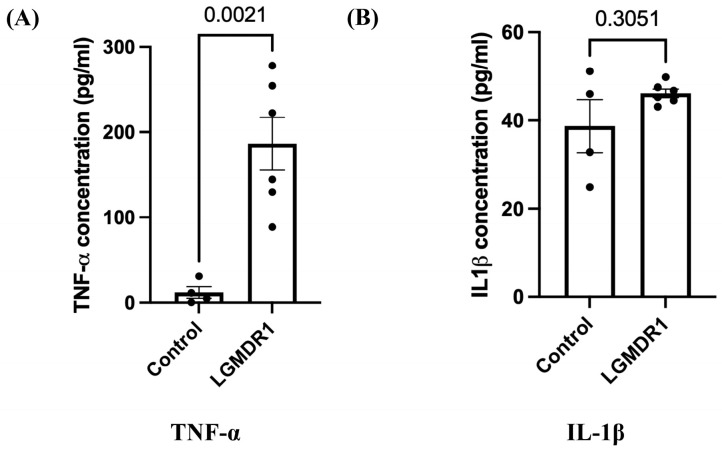
Expression of TNF-α and IL-1β cytokines in LGMDR1 patients by ELISA. (**A**) Column bar graph showing significant upregulation of TNF-α cytokine in LGMDR1 patients (*p* = 0.0021, unpaired *t*-test with Welch’s correction). Data are mean ± SEM and shown as fold change relative to control (LGMDR1, *n* = 6; controls, *n* = 4). (**B**) Column bar graph showing upregulation of IL-1β cytokine in LGMDR1 patients (*nsp* = 0.3051, unpaired *t*-test with Welch’s correction). Data are mean ± SEM and shown as fold change relative to control (LGMDR1, *n* = 6; controls, *n* = 4).

ELISA data (using serum samples) showed a significant difference in the level of TNF-α (Figure 4A, *p* = 0.0021, unpaired *t*-test with Welch’s correction; 95% CI: 94.83–254.0) in LGMDR1 patients (*n* = 6) as compared to controls (*n* = 4). There was no significant difference in the level of IL-1β (Figure 4B, *p* = 0.3051, unpaired *t*-test with Welch’s correction; 95% CI: −11.42–26.33) in LGMDR1 patients compared to control subjects. The values of IL-6 were not calculated as they were below the detection limit of the kit, except for two patients.

### 3.3. Expression of Transcription Factors in Muscle Biopsies of Calpainopathy Patients

**Figure 5:**
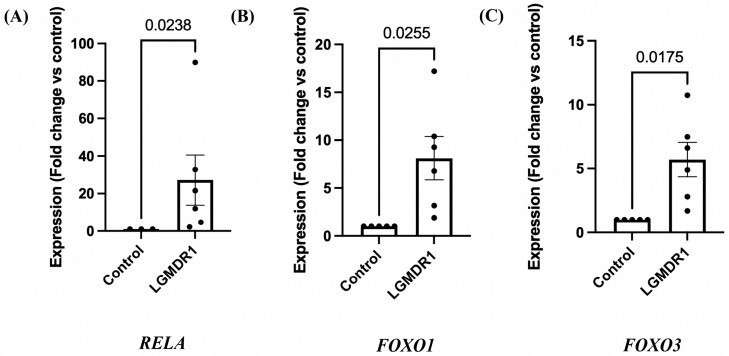
Expression of *RELA*, *FOXO1*, and *FOXO3* genes in LGMDR1 patients by qRT-PCR. (**A**) Column bar graph showing significant upregulation of *RELA* gene expression in LGMDR1 patients (*p* = 0.0238, Mann–Whitney test). Data are mean ± SEM and shown as fold change relative to control (LGMDR1, *n* = 6; controls, *n* = 3). (**B**) Column bar graph showing significant upregulation of *FOXO1* gene expression in LGMDR1 patients (*p* = 0.0255, unpaired *t*-test with Welch’s correction). Data are mean ± SEM and shown as fold change relative to control (LGMDR1, *n* = 6; controls, *n* = 5). (**C**) Column bar graph showing significant upregulation of *FOXO3* gene expression in LGMDR1 patients (*p* = 0.0175, unpaired *t*-test with Welch’s correction). Data are mean ± SEM and shown as fold change relative to control (LGMDR1, *n* = 6; controls, *n* = 5).

The gene expression for *RELA, FOXO1,* and *FOXO3* transcription factors was analyzed by qRT-PCR in LGMDR1 patients (*n* = 6). Significant upregulation of genes *RELA* (Figure 5A, *p* = 0.0238, Mann–Whitney test) was observed in patients as compared to controls (*n* = 3). Additionally, *FOXO1* (Figure 5B, *p* = 0.0255, unpaired *t*-test with Welch’s correction; 95% CI: 1.299–12.93) was observed in patients as compared to controls. Increased expression of the *FOXO3* gene (Figure 5C, *p* = 0.0175, unpaired *t*-test with Welch’s correction; 95% CI: 1.240–8.174) was also significant in LGMDR1 patients compared to controls (*n* = 5).

**Figure 6:**
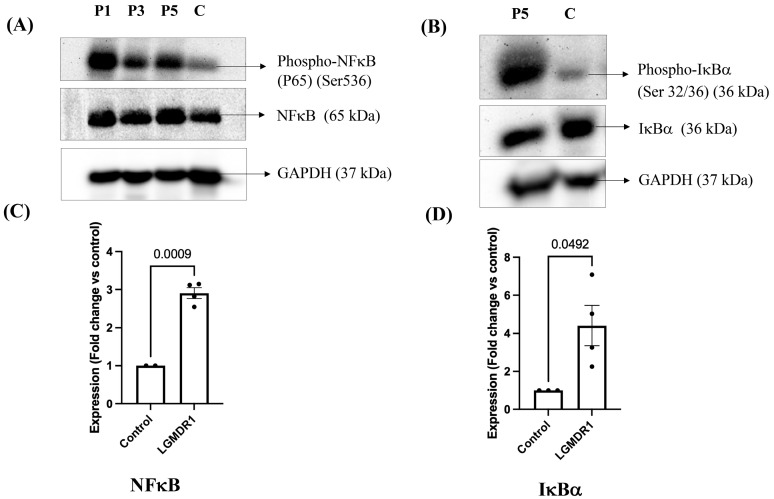
Expression of NFκB and IκBα proteins in LGMDR1 patients by Western blotting. (**A**) Phospho-NFκB (P65) and NFκB (P65) protein expression in LGMDR1 patients by Western blot. (**B**) Phospho-IκBα and IκBα protein expression in LGMDR1 patients by Western blot. C, control; P1, P3, P5, individual LGMDR1 patient samples (**C**) Column bar graph showing significant upregulation of NFκB protein expression in LGMDR1 patients (*p* = 0.0009, unpaired *t*-test with Welch’s correction). Data are mean ± SEM and shown as fold change relative to control (LGMDR1, *n* = 4; controls, *n* = 2). (**D**) Column bar graph showing upregulation of IκBα protein expression in LGMDR1 patients (*p* = 0.0492, unpaired *t*-test with Welch’s correction). Data are mean ± SEM and shown as fold change relative to control (LGMDR1, *n* = 4; controls, *n* = 3).

Out of six patients, four patients showed increased phosphorylation of NFκB protein at 65 kDa band position (Western blot; Figure 6A) as compared to controls (*n* = 2), and the observation was statistically significant (Figure 6C, *p* = 0.0009, unpaired *t*-test with Welch’s correction; 95% CI: 1.457–2.355). IκBα protein expression was analyzed by Western blot in these 4 LGMDR1 patients where phosphorylation of NFκB was increased; these patients also showed increased phosphorylation of IκBα at 36 kDa band position (Figure 6B) compared to controls (*n* = 3); the observation was statistically significant *p* = 0.0492 (Figure 6D, unpaired *t*-test with Welch’s correction; 95% CI: 0.02329–6.789).

**Figure 7:**
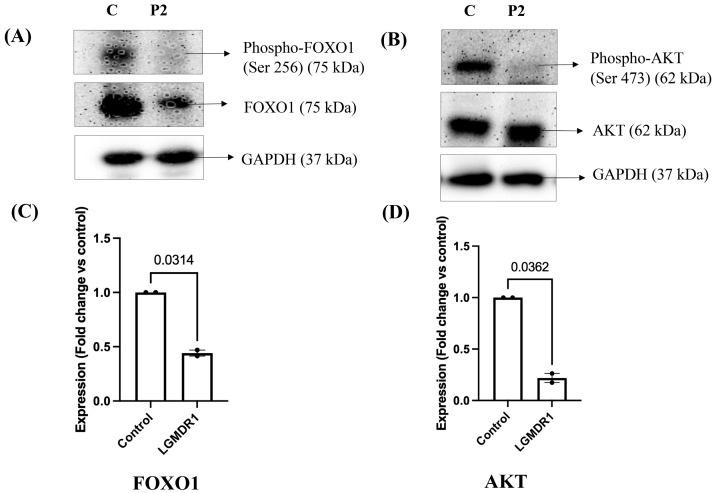
Expression of FOXO1 and AKT proteins in LGMDR1 patients by Western blotting. (**A**) Phospho-FOXO1 and FOXO1 protein expression by Western blot. (**B**) Phospho-AKT and AKT protein expression by Western blot. C, control; P2, individual LGMDR1 patient sample (**C**) Column bar graph showing decreased phosphorylation of FOXO1 protein in LGMDR1 patients (*p* = 0.0314, unpaired *t*-test with Welch’s correction). Data are mean ± SEM and shown as fold change relative to control (LGMDR1, *n* = 2; controls, *n* = 2). (**D**) Column bar graph showing significantly decreased phosphorylation of AKT protein in LGMDR1 patients (*p* = 0.0362, unpaired *t*-test with Welch’s correction). Data are mean ± SEM and shown as fold change relative to control (LGMDR1, *n* = 2; controls, *n* = 2).

FOXO1 protein expression was also analyzed, showing decreased phosphorylation (Ser 256) at the 75 kDa band position (Figure 7A) in 2 out of 6 LGMDR1 patients compared to control subjects (*n* = 2). This observation was statistically significant, *p* = 0.0314 (Figure 7C, unpaired *t*-test with Welch’s correction; 95% CI: −0.9091 to −0.2081). The expression of AKT protein, which is an inhibitory factor of FOX protein expression, was additionally analyzed in these 2 LGMDR1 patients, where phosphorylated FOXO1 protein expression was reduced. As seen in Figure 7B, LGMDR1 patients showed decreased phosphorylation of AKT protein (Ser 473) at 62 kDa position as compared to control subjects (*n* = 2), and this observation was also statistically significant, *p* = 0.0362 (Figure 7D, unpaired *t*-test with Welch’s correction; 95% CI: −1.346 to −0.2164).

## 4. Discussion

The molecular mechanism with reference to muscle atrophy in LGMDR1 patients is not fully elucidated. Histopathological features of calpainopathy patients indicate that inflammation may contribute to the muscle loss observed in these patients. Previous studies have shown that pro-inflammatory cytokines TNF-α, IL-1, and IL-6 cause muscle atrophy in mice [27]. Additionally, chronic inflammatory state, mitochondrial dysfunction, and protein turnover collectively enhance skeletal muscle loss [45]. However, there is no study on muscle atrophy of LGMDR1 patients with a background of inflammation. To our knowledge, this is the first study to investigate cytokine levels in LGMDR1 patient serum and explore their association with UPS markers and muscle atrophy. Further, the role of signaling pathways in the context of increased inflammatory cytokines in these patients is poorly understood.

**Figure 8:**
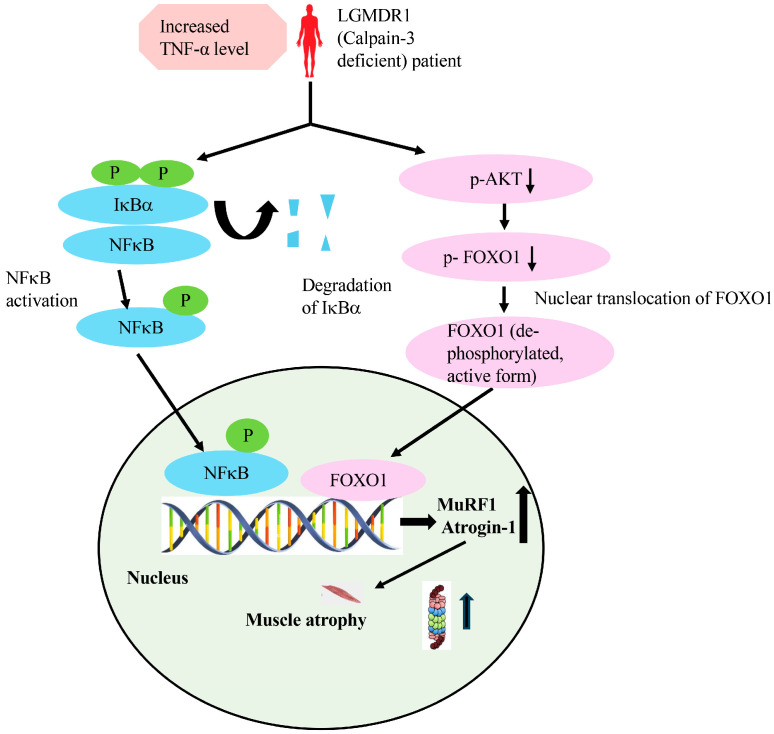
Overview of inflammation-induced muscle atrophy in LGMDR1. Inflammation may play an essential role in driving muscle atrophy in LGMDR1 patients. The current study showed a significant increase in the expression of pro-inflammatory cytokines—*TNF-α*, *IL-1β*, and *IL-6* in muscle biopsies of LGMDR1 patients, as measured by qRT-PCR. Among these, only TNF-α expression was significantly increased in muscle tissue (qRT-PCR) and serum samples (by ELISA) of these patients, compared to controls. However, IL-1β level was not statistically significant in these patients. Additionally, IL-6 level was not significantly detectable in serum samples of these patients. This may be because of a lack of correlation between mRNA and protein abundance due to post-transcriptional regulation. Thus, the data suggest that TNF-α is a muscle-wasting factor in LGMDR1 patients. Blue indicates the NFκB pathway, pink indicates the AKT/FOXO signaling pathway, “P” denotes phosphorylation, and “p-” indicates the phosphorylated form of the indicated protein.

Figure 8 summarizes the data from the current study; our data show significantly increased levels of TNF-α expression in LGMDR1 patients, with an increase in muscle-specific ubiquitin ligases (MuRF1 and Atrogin-1), suggestive of activation of the UPS pathway, which correlated with an increase in round atrophic fibers as histopathological features, along with progressive proximal muscle weakness in the patients. It may therefore be inferred that increased expression of these ubiquitin ligases (MuRF1 and Atrogin-1) may cause increased protein ubiquitinylation (transfer of an activated ubiquitin molecule to the substrates), resulting in increased muscle protein degradation through the 26S proteasome complex. Consistent with our findings, a recent study by Kanai et al. has highlighted that inflammatory cytokine-induced muscle loss activates downstream NFκB signaling that enhances Atrogin-1 and MuRF1 [46].

A recent study by Liu et al. has shown that MuRF1 is broadly considered a key atrogene for skeletal muscle loss through UPS activation [47]. This signaling cascade contributes to progressive muscle fiber loss characteristic of LGMDR1. Similarly, a previous study reported that activation of intracellular degenerative pathways contributes to progressive muscle atrophy and pathological changes in calpainopathy [48]. Our observation was similar to the study by Rajakumar et al., 2013, where both MuRF1 and Atrogin-1 protein levels were increased in calpainopathy patients [34]. The study by Rajakumar et al. (2013) highlights that oxidative stress activates NFκB, which in turn enhances the activity of the ubiquitin–proteasome pathway by increasing the expression of both MuRF1 and Atrogin-1 [34]. In contrast, Fanin M. et al. (2013) reported only increased MuRF1 expression, whereas there was no increase in Atrogin-1 protein in LGMDR1 patients, suggesting that MuRF1 is a biomarker for muscle atrophy [17]. According to Fanin et al. (2013), the differential expression of MuRF1 and Atrogin-1 could be associated with their modulation by different biological pathways [17].

Currently, there is no study on the role of the NFκB pathway in the muscle wasting of LGMDR1 patients with a background of inflammatory cytokines. A significantly increased expression of NFκB was observed at the gene and protein levels in LGMDR1 patients compared to the control group. Increased phosphorylation of NFκB (P65) protein at serine 536 was observed in 4 out of 6 patients, implying a 67% positive correlation of NFκB phosphorylation in these patients. Further support for NFκB activation was confirmed by the increased phosphorylation of IκBα at serine 32/36 in the same subset of patients. These observations suggested that IκBα is phosphorylated and degraded through the proteasome machinery. This condition allows free NFκB to enter the nucleus, activating downstream target genes—*MuRF1* and Atrogin-1—key regulators of muscle protein breakdown via the ubiquitin–proteasome pathway. The recent literature has shown that activation of the NFκB pathway in different cell types enhances inflammation, macrophage accumulation, reduced regeneration, and muscle loss, thereby maintaining a continuous inflammatory state during muscle regeneration [49]. Additionally, the NFκB signaling pathway and mitochondrial dysfunction have shown a mechanistic link to skeletal muscle atrophy, further highlighting the contribution of inflammatory-catabolic signaling in muscle loss [50]. Our finding is consistent with the previous literature suggesting increased expression of NFκB p65 under oxidative stress in LGMDR1 patients, resulting in upregulated protein ubiquitinylation [34].

While the implication of the AKT–FOXO signaling pathway in muscle atrophy has been proven in other conditions, its contribution in calpainopathy patients, specifically in the context of inflammation, remains completely unexplored. Skeletal muscle mass is controlled by various signaling pathways that maintain the balance between protein synthesis and breakdown, and impairment of this balance can lead to muscle atrophy [51]. Data from the present study provide initial evidence highlighting that dysregulation of the AKT–FOXO axis may result in muscle fiber loss in LGMDR1 patients. Specifically, we found an increased expression of *FOXO1* and *FOXO3* genes in muscle biopsies from LGMDR1 patients. These transcription factors are well-known in muscle catabolism and can increase the expression of Atrogin-1 and MuRF1. The data showed that 2 of 6 patients (33%) had decreased phosphorylation of FOXO1 at serine 256, along with reduced phosphorylation of the upstream AKT protein. Since AKT-mediated phosphorylation usually suppresses FOXO activity by sequestering it in the cytoplasm, reduced phosphorylation suggests increased nuclear translocation and transcriptional activity of FOXO1 (in contrast to increased phosphorylation of AKT and FOXO1 proteins observed in control subjects). Experimental models of muscle atrophy have similarly highlighted upregulated NFκB signaling together with reduced FOXO phosphorylation, further supporting elevated catabolic signaling during muscle degeneration [52]. As our sample size is small, additional studies with larger patient numbers will provide deeper insight into this mechanism. Recent reviews have suggested that inflammation, mitochondrial dysfunction, and mechanical stress activate pathways such as FOXO signaling, the ubiquitin–proteasome system, and autophagy, which promote skeletal muscle atrophy [53]. Our findings demonstrate that impaired AKT signaling cannot inhibit FOXO proteins, allowing their activation and subsequent induction of Atrogin-1 and MuRF1 (E3 ligases), resulting in muscle atrophy by activating the UPS pathway.

This study has some limitations. First, the sample size was limited; therefore, future studies involving larger patient cohorts will be needed to further extend these findings. Due to the limited sample size, no significant correlation was observed between MuRF1 or Atrogin-1 expression and CK levels. Correlation with other clinical or histopathological features was not performed. Future studies with larger cohorts will help to establish these relationships. Second, immunostaining for TNFR1 and TNFR2 could not be performed due to insufficient remaining muscle biopsy tissue. Despite these limitations, the present findings provide mechanistic insight into inflammation-associated muscle atrophy in LGMDR1. Given the limited sample size and multiple endpoints analyzed, the findings of this study should be interpreted as exploratory.

The present study highlights that inflammatory conditions in LGMDR1 patients upregulate expression of both MuRF1 and Atrogin-1 (E3 ligases), suggesting increased protein ubiquitination and degradation of muscle protein. Increased expression of TNF-α is associated with markers of muscle wasting in LGMDR1 patients. The current study provides insight into a possible mechanism linking inflammatory signaling with increased NFκB activity and alterations in the AKT–FOXO axis, which may contribute to the activation of MuRF1 and Atrogin-1, key regulators of muscle atrophy in LGMDR1 patients.

## 5. Conclusions

The present study demonstrates the inflammation-linked muscle atrophy in LGMDR1 patients through activation of the NFκB and reduced AKT-FOXO activity. The observed dysregulation of the two signaling pathways highlights mechanisms implying disease etiology and broadens our understanding of targeting inflammatory signaling in future therapeutic strategies. Our observations support the involvement of TNF-α–linked UPS dysregulation in LGMDR1 patients; however, further studies are required to discover treatments that decrease inflammation or target specific signaling pathways to prevent muscle protein breakdown.

## Data Availability

The data presented in this study are available on request from the corresponding author due to ethical restrictions related to patient confidentiality.

## Supplementary Materials

The following supporting information can be downloaded at: https://www.mdpi.com/article/10.3390/cimb48040361/s1.

## Abbreviations

MuRF1	Muscle RING-finger protein-1
TNF-α	Tumor necrosis factor alpha
IL-1β	Interleukin 1-beta
IL-6	Interleukin 6
NFκB	Nuclear factor-kappa B
IκBα	Nuclear factor-kappa B inhibitor alpha
FOXO	Forkhead box transcription factors
TRIM63	Tripartite motif-containing protein 63
FBXO32	F-box protein 32
AKT	Protein Kinase B

## References

[1] Richard I., Broux O., Allamand V., Fougerousse F., Chiannilkulchai N., Bourg N., Brenguier L., Devaud C., Pasturaud P., Roudaut C. (1995). Mutations in the proteolytic enzyme calpain 3 cause limb-girdle muscular dystrophy type 2A. Cell.

[2] Shin J.-H., Kim H.-S., Lee C.-H., Kim C.-M., Park K.-H., Kim D.-S. (2007). Mutations of CAPN3 in Korean patients with limb-girdle muscular dystrophy. J. Korean Med. Sci..

[3] Liewluck T. (2025). Limb-girdle muscular dystrophies. Continuum.

[4] Zhong H., Zheng Y., Zhao Z., Lin P., Xi J., Zhu W., Lin J., Lu J., Yu M., Zhang W. (2021). Molecular landscape of CAPN3 mutations in limb-girdle muscular dystrophy type R1: From a Chinese multicentre analysis to a worldwide perspective. J. Med. Genet..

[5] Zhang L., Zhang Y., Han C., Jiang J., Jiang J., Cai X., Yu L., Qi H., Fang Q., Ding D. (2024). Two novel variants of the *CAPN3* gene in Chinese patients with limb-girdle muscular dystrophy recessive 1. Hum. Hered..

[6] Bardakov S.N., Sorochanu I., Mkrtchyan L.A., Slesarenko Y.S., Tsargush V.A., Limaev I.S., Isaev A.A., Yakovlev I.A., Deev R.V. (2025). Calpainopathy (limb-girdle muscular dystrophy type R1): Clinical features, diagnostic approaches, and biotechnological treatment methods. J. Neuromuscul. Dis..

[7] Şahin İ.O., Özkul Y., Dündar M. (2021). Current and future therapeutic strategies for limb girdle muscular dystrophy type R1: Clinical and experimental approaches. Pathophysiology.

[8] Magri F., Del Bo R., D’Angelo M.G., Sciacco M., Gandossini S., Govoni A., Napoli L., Ciscato P., Fortunato F., Brighina E. (2012). Frequency and characterisation of anoctamin 5 mutations in a cohort of Italian limb-girdle muscular dystrophy patients. Neuromuscul. Disord..

[9] Zídková J., Kramářová T., Kopčilová J., Réblová K., Haberlová J., Mazanec R., Voháňka S., Gřegořová A., Langová M., Honzík T. (2023). Genetic findings in Czech patients with limb girdle muscular dystrophy. Clin. Genet..

[10] Rico A., Guembelzu G., Palomo V., Martínez A., Aiastui A., Casas-Fraile L., Valls A., López de Munain A., Sáenz A. (2021). Allosteric modulation of GSK-3β as a new therapeutic approach in limb girdle muscular dystrophy R1 calpain 3-related. Int. J. Mol. Sci..

[11] Sáenz A., Leturcq F., Cobo A.M., Poza J.J., Ferrer X., Otaegui D., Camaño P., Urtasun M., Vílchez J., Gutiérrez-Rivas E. (2005). LGMD2A: Genotype–phenotype correlations based on a large mutational survey on the calpain 3 gene. Brain.

[12] Shinkai-Ouchi F., Itoh Y., Shindo M., Mikami K., Iguchi Y., Hata S., Tsutsumi R., Izumi-Mishima Y., Machida K., Suzuki Y. (2025). Distinct systemic metabolic features in limb-girdle muscular dystrophy type R1 mouse models as a potential early pathogenic signature. Biochim. Biophys. Acta Mol. Basis Dis..

[13] Becker N., Moore S.A., Jones K.A. (2022). The inflammatory pathology of dysferlinopathy is distinct from calpainopathy, Becker muscular dystrophy, and inflammatory myopathies. Acta Neuropathol. Commun..

[14] Ozyilmaz B., Kirbiyik O., Ozdemir T.R., Ozer O.K., Kutbay Y.B., Erdogan K.M., Guvenc M.S., Arıkan Ş., Turk T.S., Kale M.Y. (2022). Experiences in the molecular genetic and histopathological evaluation of calpainopathies. Neurogenetics.

[15] D’Este G., Giorgetti A., Cassandrini D., Magri F., Ronchi D., Rubegni A., Lopergolo D., Malandrini A., Merlini L., Vattemi G. (2025). Recurrent *CAPN3* p.Asp753Asn Variant Supports a Potential Dominant Calpainopathy with Variable Clinical Expressivity. Int. J. Mol. Sci..

[16] Campuzano-Donoso M., Reytor-González C., Toral-Noristz M., González Y., Simancas-Racines D. (2026). Molecular Bases of Myopathies and Their Impact on Clinical Practice: Advances and Future Perspectives. Int. J. Mol. Sci..

[17] Fanin M., Nascimbeni A.C., Angelini C. (2013). Muscle atrophy in Limb Girdle Muscular Dystrophy 2A: A morphometric and molecular study. Neuropathol. Appl. Neurobiol..

[18] Kim J.T., Jeon D.H., Lee H.J. (2024). Molecular Mechanism of Skeletal Muscle Loss and Its Prevention by Natural Resources. Food Sci. Biotechnol..

[19] Fanzani A., Conraads V.M., Penna F., Martinet W. (2012). Molecular and cellular mechanisms of skeletal muscle atrophy: An update. J. Cachexia Sarcopenia Muscle.

[20] Pang X., Zhang P., Chen X., Liu W. (2023). Ubiquitin-Proteasome Pathway in Skeletal Muscle Atrophy. Front. Physiol..

[21] Singh A., Yadav A., Phogat J., Dabur R. (2022). Dynamics and Interplay between Autophagy and Ubiquitin–Proteasome System Coordination in Skeletal Muscle Atrophy. Curr. Mol. Pharmacol..

[22] Dong H., Lyu Y., Huang C.Y., Tsai S.Y. (2025). Limiting Cap-Dependent Translation Increases 20S Proteasomal Degradation and Protects the Proteomic Integrity in Autophagy-Deficient Skeletal Muscle. Autophagy.

[23] Brooks P., Fuertes G., Murray R.Z., Bose S., Knecht E., Rechsteiner M.C., Hendil K.B., Tanaka K., Dyson J., Rivett J. (2000). Subcellular localization of proteasomes and their regulatory complexes in mammalian cells. Biochem. J..

[24] Grice G.L., Nathan J.A. (2016). The recognition of ubiquitinated proteins by the proteasome. Cell. Mol. Life Sci..

[25] Souza A.L., Alves A.L., Martinez C.G., Sousa J.C., Kurtenbach E. (2025). Biomarkers of Skeletal Muscle Atrophy Based on Atrogenes Evaluation: A Systematic Review and Meta-Analysis Study. Int. J. Mol. Sci..

[26] Hirose T., Nakazato K., Song H., Ishii N. (2008). TGF-β1 and TNF-α are involved in the transcription of type I collagen α2 gene in soleus muscle atrophied by mechanical unloading. J. Appl. Physiol..

[27] Andrianjafiniony T., Dupré-Aucouturier S., Letexier D., Couchoux H., Desplanches D. (2010). Oxidative stress, apoptosis, and proteolysis in skeletal muscle repair after unloading. Am. J. Physiol. Cell Physiol..

[28] Caron A.Z., Drouin G., Desrosiers J., Trensz F., Grenier G. (2009). A novel hindlimb immobilization procedure for studying skeletal muscle atrophy and recovery in mouse. J. Appl. Physiol..

[29] Ghosh S., May M.J., Kopp E.B. (1998). NF-κB and Rel proteins: Evolutionarily conserved mediators of immune responses. Annu. Rev. Immunol..

[30] Cai D., Frantz J.D., Tawa N.E., Melendez P.A., Oh B.-C., Lidov H.G.W., Hasselgren P.-O., Frontera W.R., Lee J., Glass D.J. (2004). IKKβ/NF-κB activation causes severe muscle wasting in mice. Cell.

[31] Adams V., Mangner N., Gasch A., Krohne C., Gielen S., Hirner S., Thierse H.-J., Witt C.C., Linke A., Schuler G. (2008). Induction of MuRF1 is essential for TNF-α-induced loss of muscle function in mice. J. Mol. Biol..

[32] Judge A.R., Koncarevic A., Hunter R.B., Liou H.C., Jackman R.W., Kandarian S.C. (2007). Role for IκBα, but not c-Rel, in skeletal muscle atrophy. Am. J. Physiol. Cell Physiol..

[33] Kramerova I., Kudryashova E., Wu B., Germain S., Vandenborne K., Romain N., Haller R.G., Verity M.A., Spencer M.J. (2009). Mitochondrial abnormalities, energy deficit and oxidative stress are features of calpain 3 deficiency in skeletal muscle. Hum. Mol. Genet..

[34] Rajakumar D., Alexander M., Oommen A. (2013). Oxidative stress, NF-κB and the ubiquitin proteasomal pathway in the pathology of calpainopathy. Neurochem. Res..

[35] Comim C.M., Mathia G.B., Hoepers A., Tuon L., Kapczinski F., Dal-Pizzol F., Quevedo J., Rosa M.I. (2015). Neurotrophins, cytokines, oxidative parameters and functionality in progressive muscular dystrophies. An. Acad. Bras. Cienc..

[36] Fanin M., Angelini C. (2015). Protein and genetic diagnosis of limb girdle muscular dystrophy type 2A: The yield and the pitfalls. Muscle Nerve.

[37] Katoh M., Katoh M. (2004). Human FOX gene family. Int. J. Oncol..

[38] Brunet A., Bonni A., Zigmond M.J., Lin M.Z., Juo P., Hu L.S., Anderson M.J., Arden K.C., Blenis J., Greenberg M.E. (1999). Akt promotes cell survival by phosphorylating and inhibiting a Forkhead transcription factor. Cell.

[39] Brunet A., Kanai F., Stehn J., Xu J., Sarbassova D., Frangioni J.V., Dalal S.N., DeCaprio J.A., Greenberg M.E., Yaffe M.B. (2002). 14-3-3 transits to the nucleus and participates in dynamic nucleocytoplasmic transport. J. Cell Biol..

[40] Obsilova V., Vecer J., Herman P., Pabianova A., Sulc M., Teisinger J., Boura E., Obsil T. (2005). 14-3-3 protein interacts with nuclear localization sequence of forkhead transcription factor FoxO4. Biochemistry.

[41] Sandri M., Sandri C., Gilbert A., Skurk C., Calabria E., Picard A., Walsh K., Schiaffino S., Lecker S.H., Goldberg A.L. (2004). Foxo transcription factors induce the atrophy-related ubiquitin ligase atrogin-1 and cause skeletal muscle atrophy. Cell.

[42] Stitt T.N., Drujan D., Clarke B.A., Panaro F., Timofeyva Y., Kline W.O., Gonzalez M., Yancopoulos G.D., Glass D.J. (2004). The IGF-1/PI3K/Akt pathway prevents expression of muscle atrophy-induced ubiquitin ligases by inhibiting FOXO transcription factors. Mol. Cell.

[43] Banerjee S., Radotra B.D., Luthra-Guptasarma M., Goyal M.K. (2024). Identification of novel pathogenic variants of calpain-3 gene in limb girdle muscular dystrophy R1. Orphanet J. Rare Dis..

[44] Banerjee S., Radotra B.D. (2025). Unraveling novel pathogenic CAPN3 variants in limb-girdle muscular dystrophy R1. J. Rare Dis. Res. Treat..

[45] Cui X., Liu H., Liu Y., Yu Z., Wang D., Wei W., Wang S. (2025). The Role and Mechanisms of Myokines in Sarcopenia: New Intervention Strategies for the Challenges of Aging. Front. Med..

[46] Kanai M., Ganbaatar B., Endo I., Ohnishi Y., Teramachi J., Tenshin H., Higa Y., Hiasa M., Mitsui Y., Hara T. (2024). Inflammatory Cytokine-Induced Muscle Atrophy and Weakness Can Be Ameliorated by an Inhibition of TGF-β-Activated Kinase-1. Int. J. Mol. Sci..

[47] Liu X., Wen Y., Lu Y. (2024). Targeting MuRF1 to combat skeletal muscle wasting in cardiac cachexia: Mechanisms and therapeutic prospects. Med. Sci. Monit. Int. Med. J. Exp. Clin. Res..

[48] Angelini C., Tasca E., Nascimbeni A.C., Fanin M. (2014). Muscle fatigue, nNOS and muscle fiber atrophy in limb girdle muscular dystrophy. Acta Myol..

[49] Pryce B.R., Oles A., Talbert E.E., Romeo M.J., Vaena S., Sharma S., Spadafora V., Tolliver L., Mahvi D.A., Morgan K.A. (2024). Muscle Inflammation Is Regulated by NF-κB from Multiple Cells to Control Distinct States of Wasting in Cancer Cachexia. Cell Rep..

[50] Liu D., Wang S., Liu S., Wang Q., Che X., Wu G. (2024). Frontiers in Sarcopenia: Advancements in Diagnostics, Molecular Mechanisms, and Therapeutic Strategies. Mol. Asp. Med..

[51] Joshi A.S., Tomaz da Silva M., Kumar S., Kumar A. (2025). Signaling Networks Governing Skeletal Muscle Growth, Atrophy, and Cachexia. Skelet. Muscle.

[52] Kamal K.Y., Othman M.A., Kim J.H., Lawler J.M. (2024). Bioreactor Development for Skeletal Muscle Hypertrophy and Atrophy by Manipulating Uniaxial Cyclic Strain: Proof of Concept. NPJ Microgravity.

[53] Wang Y., Meng J., Zhang J., Tian L., Wei W., Tang X., Zhang Q., Ding D., Wang X., Guo Z. (2025). Cell Biomechanics on Muscle Atrophy: From Intricate Mechanisms to Therapeutic Frontiers. Ann. Med..

