# Supplementary material for: Inflammation-Linked Muscle Atrophy in Limb Girdle Muscular Dystrophy R1 (LGMDR1): Insights into Disease Mechanisms
Source: Curr Issues Mol Biol. 2026 Mar 30;48(4):361. doi: 10.3390/cimb48040361 (PMC13114790; doi:10.3390/cimb48040361)
Supplement: Supplementary file 1 [file cimb-48-00361-s001.zip › cimb-4147530-supplementary/Final Supplementary Files/Supplementary figure S1.pdf]

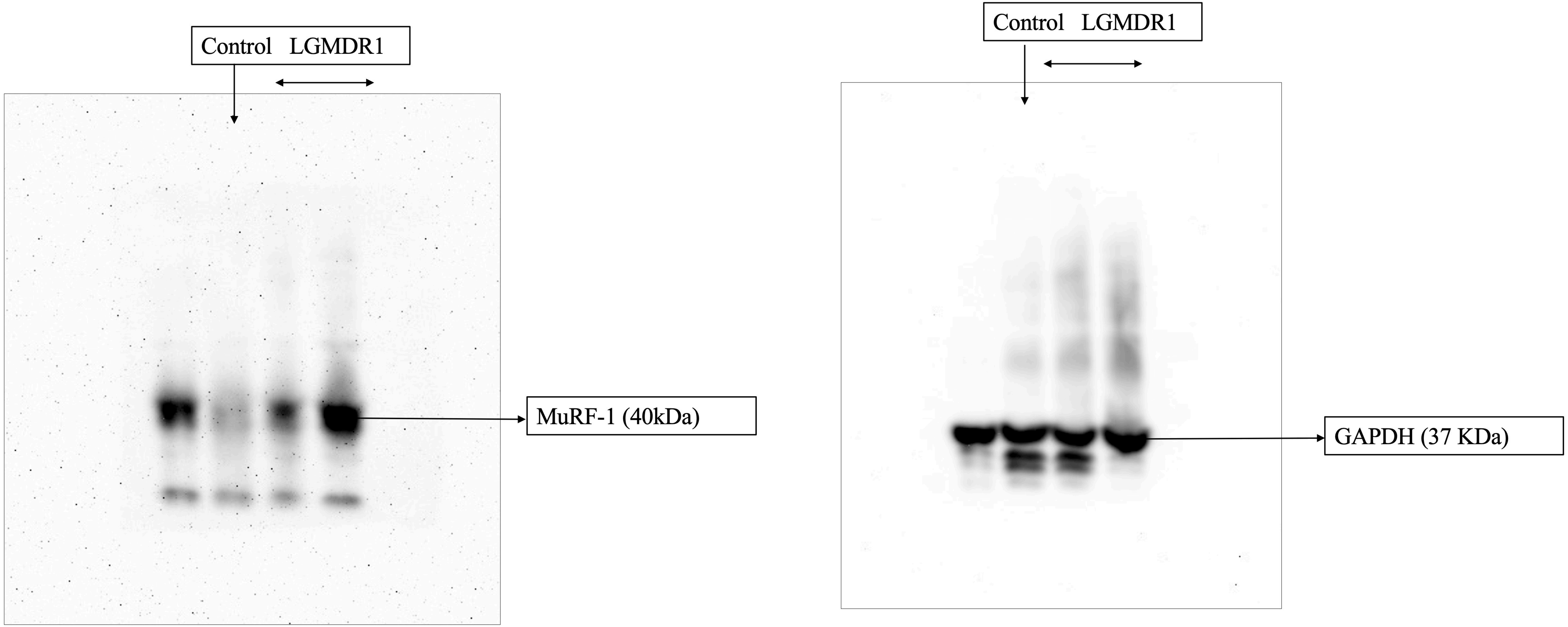

Supplementary figure 1: Expression of MuRF-1 protein in LGMDR1 patients by western blotting

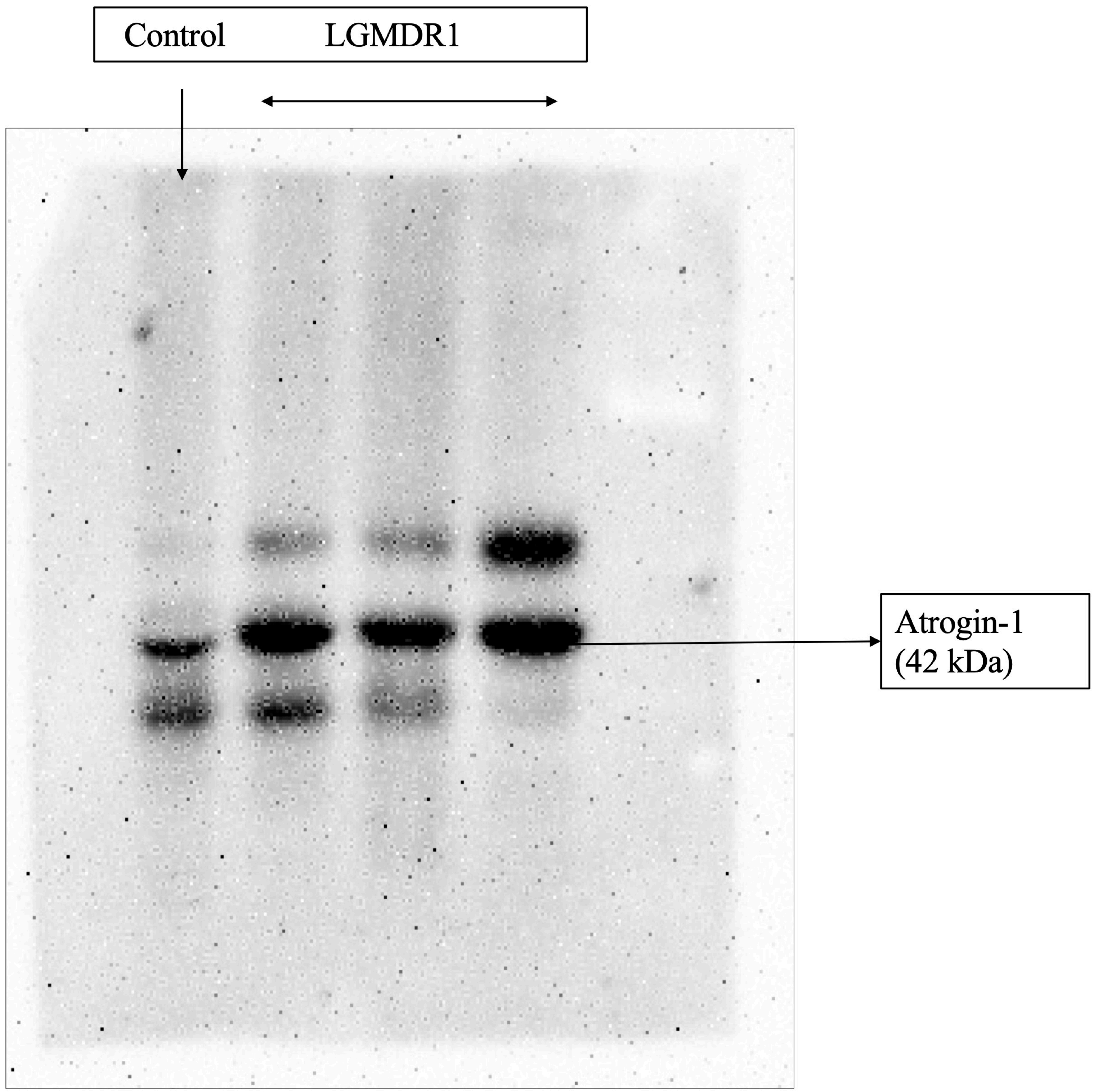

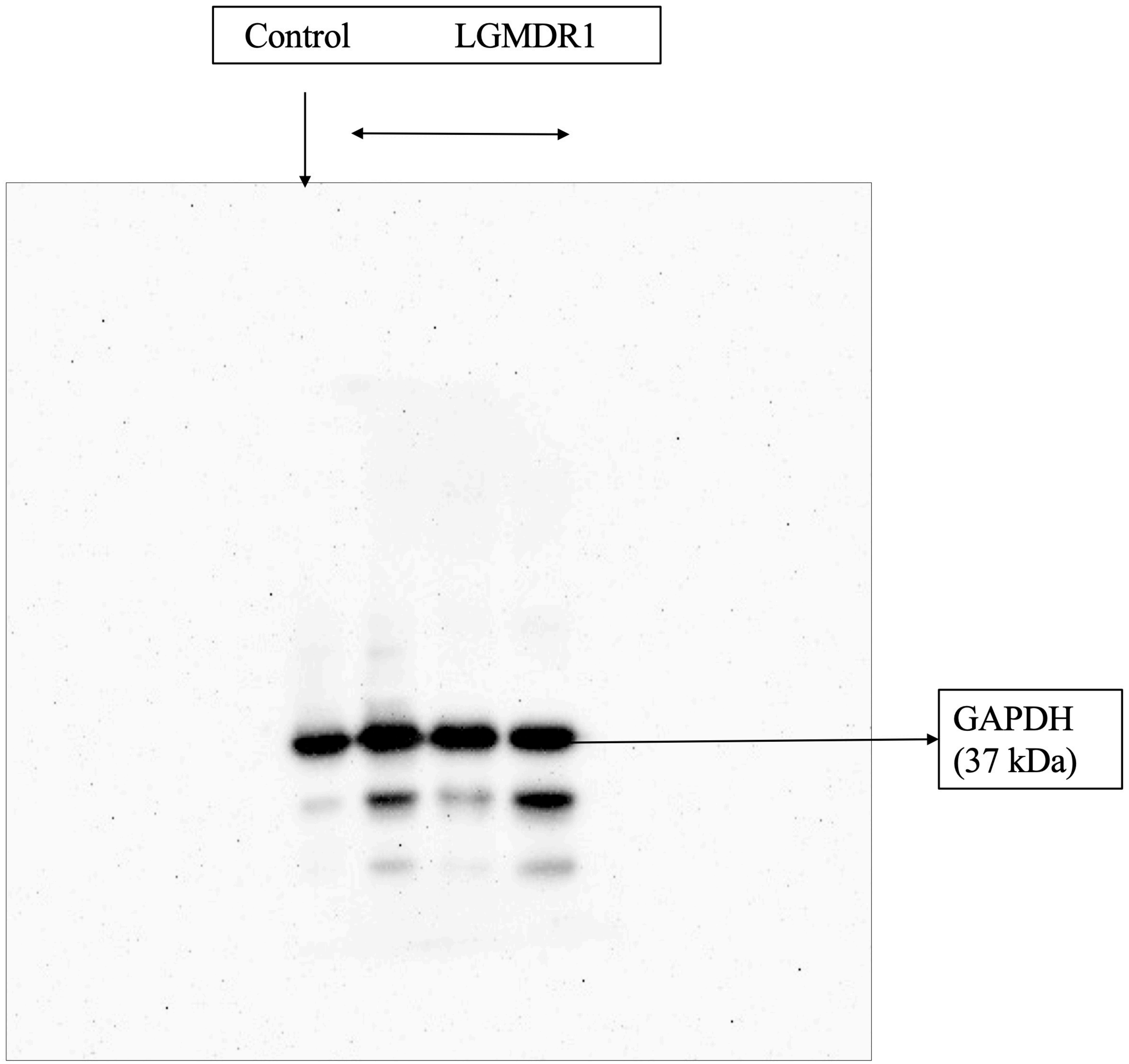

Supplementary figure 2: Expression of Atrogin-1 protein in LGMDR1 patients by western blotting

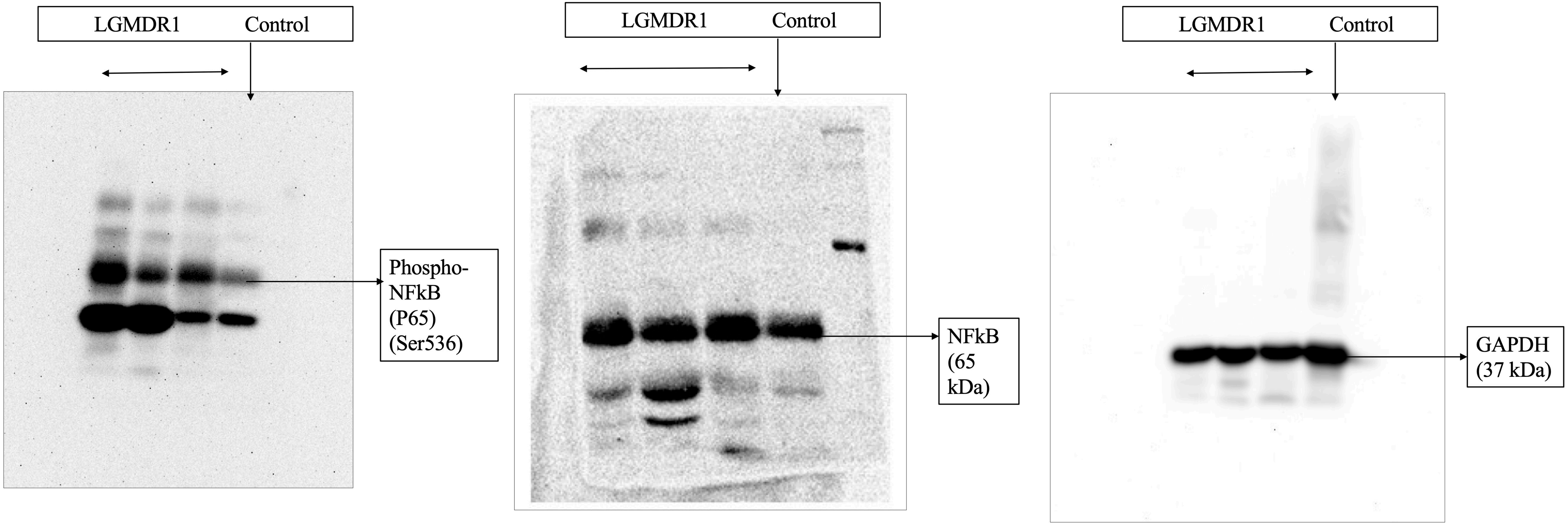

Supplementary figure 3: Expression of NFkB protein in LGMDR1 patients by western blotting

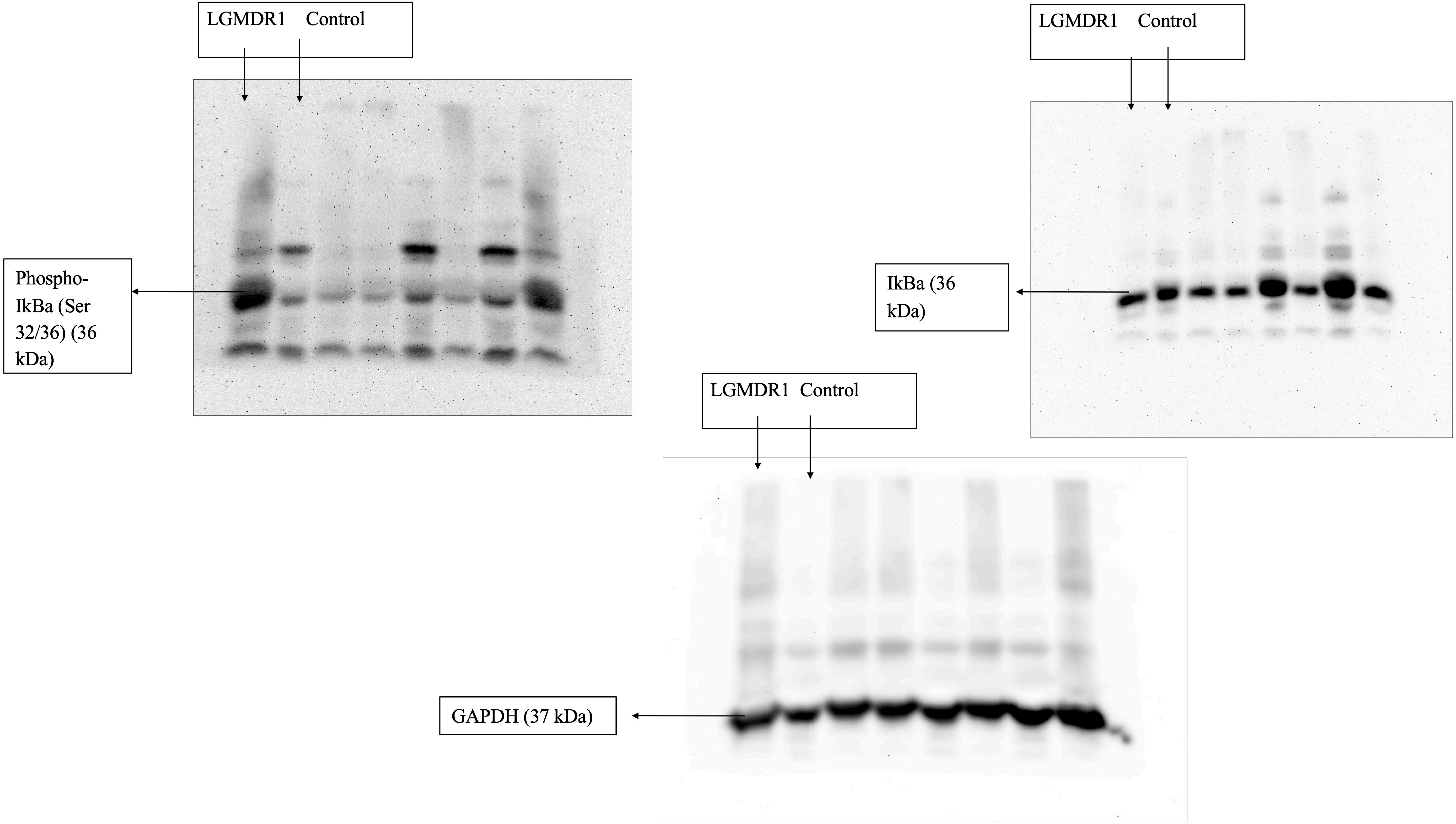

Supplementary figure 4: Expression of IkBa protein in LGMDR1 patient by western blotting

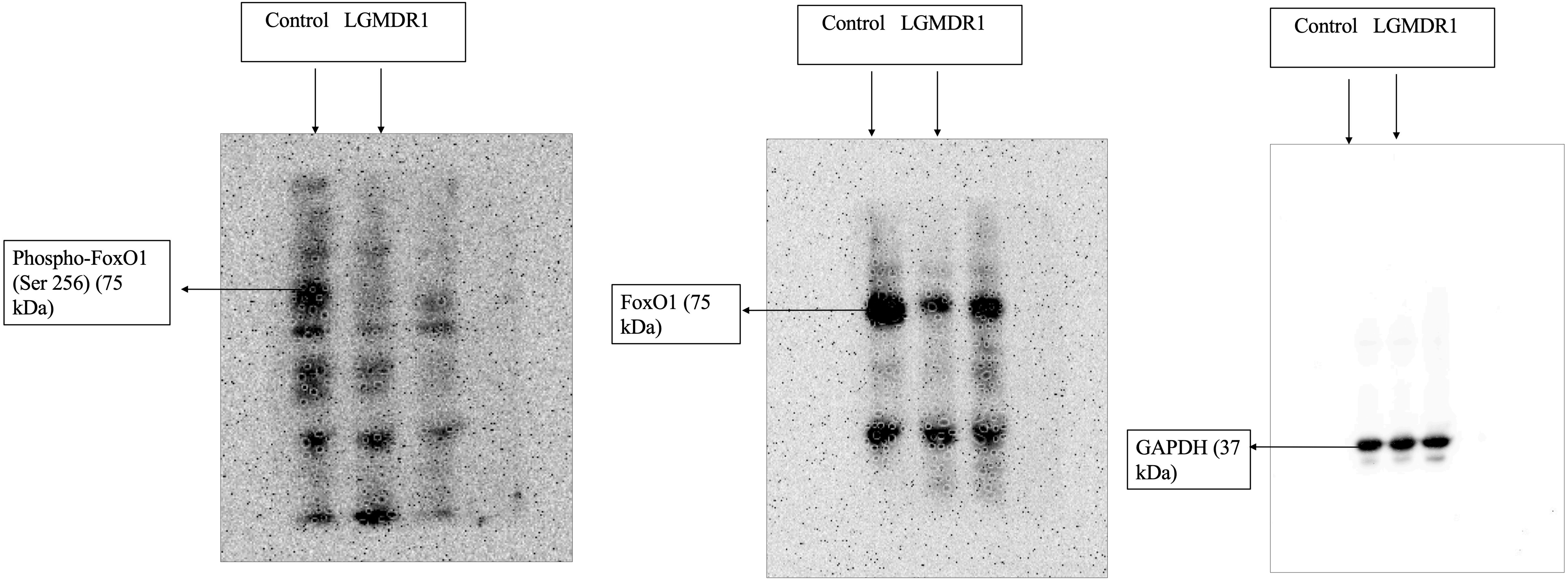

Supplementary figure 5: Expression of FoxO1 protein in LGMDR1 patient by western blotting

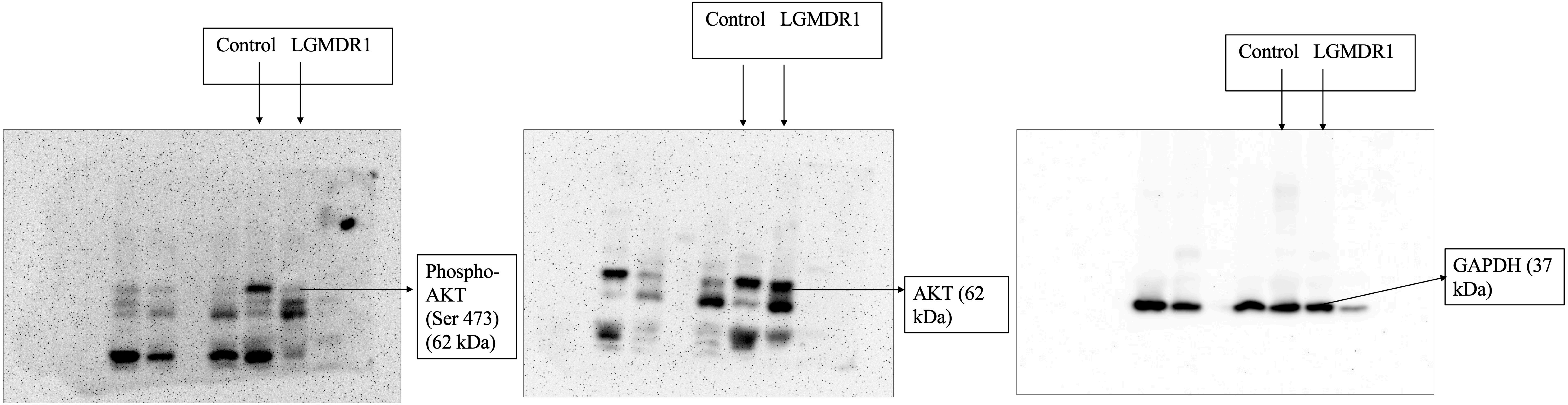

Supplementary figure 6: Expression of AKT protein in LGMDR1 patient by western blotting
