# Supplementary material for: Inflammation-Linked Muscle Atrophy in Limb Girdle Muscular Dystrophy R1 (LGMDR1): Insights into Disease Mechanisms
Source: Curr Issues Mol Biol. 2026 Mar 30;48(4):361. doi: 10.3390/cimb48040361 (PMC13114790; doi:10.3390/cimb48040361)
Supplement: Supplementary file 1 [file cimb-48-00361-s001.zip › cimb-4147530-supplementary/Final Supplementary Files/Supplementary Table S1.pdf]

Patient	Clinical features	Genetics
1	Waddling gait; wasting of biceps, triceps and supraspinatus muscle; Calf with EDB (extensor digitorum brevis) muscle and Beevor sign were observed.	c.1189T>C (Missense)
2	Waddling gait and scapular winging	c.2338G>C (Missense)
3	Proximal weakness since 2 years	c.2338G>C & c.2051-1G>T (Splice site variant & missense)
4	Problems in running since the last 6 years, difficulty in getting up	c.1688delinsTC (Insertion-deletion)
5	Difficulty in climbing stairs, getting up from squatting position. Waddling gait, scapular winging and adductor splay sign were observed	c.2338G>C (Missense)
6	Patient needed support during walking, with presence of lordotic posture and was wheelchair bound	c.1939G>T (Nonsense)

Supplementary Table 1: Clinical and genetic features of LGMDR1 patients
