# Supplementary material for: Inflammation-Linked Muscle Atrophy in Limb Girdle Muscular Dystrophy R1 (LGMDR1): Insights into Disease Mechanisms
Source: Curr Issues Mol Biol. 2026 Mar 30;48(4):361. doi: 10.3390/cimb48040361 (PMC13114790; doi:10.3390/cimb48040361)
Supplement: Supplementary file 1 [file cimb-48-00361-s001.zip › cimb-4147530-supplementary/Final Supplementary Files/Supplementary Table S2.pdf]

**Assay**	**Target**	**Patients (*n*)**	**Controls (*n*)**
qRT-PCR	*FBXO32*	6	5
qRT-PCR	*TRIM63*	6	5
Western blot	Atrogin-1	6	4
Western blot	MuRF1	6	4
qRT-PCR	*TNF*	6	5
qRT-PCR	*IL1B*	6	5
qRT-PCR	*IL6*	6	5
ELISA	TNF-α	6	4
ELISA	IL-1β	6	4
qRT-PCR	*RELA*	6	3
qRT-PCR	*FOXO1*	6	5
qRT-PCR	*FOXO3*	6	5
Western blot	NFκB	4	2
Western blot	IκBα	4	2
Western blot	FOXO1	2	2
Western blot	AKT	2	2

Supplementary Table 2: Number of samples used for each experimental analysis
